# Case Report: Dual nebulised antibiotics among adults with cystic fibrosis and chronic *Pseudomonas* infection

**DOI:** 10.12688/f1000research.13298.2

**Published:** 2018-02-28

**Authors:** Nina Mann, Shirley Murray, Zhe Hui Hoo, Rachael Curley, Martin J. Wildman

**Affiliations:** 1 Sheffield Adult Cystic Fibrosis Centre, Northern General Hospital, Sheffield, S5 7AU, UK; 2 School of Health and Related Research (ScHARR), University of Sheffield, Sheffield, S1 4DA, UK

**Keywords:** Cystic fibrosis, nebuliser therapy, pulmonary exacerbations, Pseudomonas aeruginosa

## Abstract

Pulmonary exacerbations in adults with cystic fibrosis (CF) and chronic *Pseudomonas aeruginosa* (Psae) infection are usually treated with dual intravenous antibiotics for 14 days, despite the lack of evidence for best practice. Intravenous antibiotics are commonly associated with various systemic adverse effects, including renal failure and ototoxicity. Inhaled antibiotics are less likely to cause systematic adverse effects, yet can achieve airway concentrations well above conventional minimum inhibitory concentrations. Typically one inhaled antibiotic is used at a time, but dual inhaled antibiotics (i.e. concomitant use of two different inhaled antibiotics) may have synergistic effect and achieve better results in the treatment of exacerbations. We presented anecdotal evidence for the use of dual inhaled antibiotics as an acute treatment for exacerbations, in the form of a case report. A female in her early thirties with CF and chronic Psae infection improved her FEV _1_ by 5% and 2% with two courses of dual inhaled antibiotics to treat exacerbations in 2016. In contrast, her FEV _1_ changed by 2%, –2%, 0% and 2%, respectively, with four courses of dual intravenous antibiotics in 2016. Baseline FEV _1_ was similar prior to all six courses of treatments. The greater FEV _1_ improvements with dual inhaled antibiotics compared to dual intravenous antibiotics suggest the potential role of using dual inhaled antibiotics to treat exacerbations among adults with CF and chronic Psae infection, especially since a greater choice of inhaled anti-pseudomonal antibiotics is now available. A previous study in 1985 has looked at the concomitant administration of inhaled tobramycin and carbenicillin, by reconstituting antibiotics designed for parenteral administration. To our knowledge, this is the first literature to describe the concomitant use of two different antibiotics specifically developed for delivery via the inhaled route.

## Introduction

Cystic fibrosis (CF) is a genetic condition whereby ~80% of mortalities are primarily due to lung disease ^1^. People with CF are prone to recurrent respiratory infections (termed ‘pulmonary exacerbations’), which leads to progressive lung damage and respiratory failure ^2^. This is especially so after *Pseudomonas aeruginosa* (Psae) is acquired ^3^.

Although there is a lack of evidence for best practice in treating exacerbations among adults with CF and chronic Psae infection ^4^, two weeks of dual intravenous antibiotics are generally used for synergistic effect ^4, 5^. The European CF Society recommend against using inhaled antibiotics to treat exacerbations, due to concerns that increased mucus plugs during exacerbations may prevent antibiotics from reaching smaller airways ^5^.

There is scant research on using inhaled antibiotics to treat exacerbations. A Cochrane review in 2012 found only four relevant studies, with inadequate sample sizes to demonstrate efficacy ^6^. Subsequently, a randomised cross-over trial among 20 adults with CF and pulmonary exacerbation in 2014 demonstrated longer time to next exacerbation with nebulised compared to intravenous tobramycin (all participants received intravenous colistin as the second antibiotic) ^7^. A large observational study in North America found that ~24% of exacerbations are treated with inhaled antibiotics ^8^. Inhaled antibiotics have several advantages. Systemic adverse effects e.g. allergic reactions, gastrointestinal manifestations, ototoxicity and renal failure are common with intravenous antibiotics ^9, 10^ but rare with inhaled antibiotics ^11^. Higher antibiotic concentrations within the airways are achieved via inhaled route, which may be beneficial in overcoming resistance ^12^. Inhaled route also overcomes difficulties associated with venous access.

For long-term suppressive therapy, typically one inhaled antibiotic is used at a time, and someone on multiple inhaled antibiotics would alternate between those antibiotics ^11^. Of the five existing trials looking at inhaled antibiotics for acute exacerbation, only one study with 18 participants concomitantly administed two inhaled antibiotics (tobramycin and carbenicillin) ^6^. Yet dual inhaled antibiotics (i.e. concomitant use of two different inhaled antibiotics) may have synergistic effect, thus achieve better results. We report on an adult with CF and chronic Psae infection who achieved good results when treating exacerbations using dual inhaled antibiotics.

## Case report

A Caucasian female in her early thirties with F508del/Class I mutation, pancreatic insufficiency and CF related diabetes also fulfilled the Leeds criteria ^13^ for chronic Psae infection. Only Psae (Liverpool epidemic strain sensitive only to colistin) was cultured from all 12 sputum samples in the previous year. Despite high objective adherence to nebulised dornase alfa 2.5mg once daily and alternating colistin (Promixin ^®^) 1megaunit twice daily/tobramycin (TOBI ^®^) 300mg twice daily (82.9% two years ago, 96.3% in the previous year; measured with an I-neb ^®^), stable BMI around 23.1 and reasonable glycaemic control (HbA1c 47 in month 1 of the follow-up period), there was a trend of declining %FEV _1_. Her annual best FEV _1_ was 47%, 44% and 42% over the previous three years. There was no evidence of allergic bronchopulmonary aspergillosis (ABPA) or other CF complications compromising her %FEV _1_.

In month one, Promixin ^®^ was also switched to nebulised Aztreonam (AZLI ^®^) 75mg thrice daily. She had two courses (28 days) of intravenous antibiotics throughout the previous year. She agreed to three-monthly intravenous antibiotics in the follow-up year to try halt the FEV _1_ decline. She had 14 days of home intravenous piperacillin/tazobactam 4.5g thrice daily and intravenous colistin 2megaunit thrice daily in month one (%FEV _1_ improved from 38% to 40%), and again in month 4 (%FEV _1_ declined from 40% to 38%).

She felt less well with increased sputum volume and dyspnoea at the start of month six. Her %FEV _1_ was 39%. She agreed to try 14 days of concomitant nebulised AZLI ^®^ 75mg thrice daily and TOBI ^®^ 300mg twice daily at home (nebulised dornase alfa was continued) as treatment for acute exacerbation. She used 69/70 (98.6%) of her nebuliser doses over the 14 days (note that TOBI ^®^ twice daily = 4 doses via I-neb ^®^ and AZLI ^®^ could not be administered via I-neb ^®^). Her %FEV _1_ improved to 45% at day 7 and 44% at day 14. Her symptoms resolved by day 14.

She felt well but her %FEV _1_ declined to 39% during her next clinic review at the end of month seven. She went on another 14-day course of home intravenous piperacillin/tazobactam 4.5g thrice daily and intravenous colostin 2megaunit thrice daily as treatment for acute exacerbation. At day 14, her %FEV _1_ remained 39%. Another 14-day course of home concomitant nebulised AZLI ^®^ and TOBI ^®^ was started in month 8 (nebulised dornase alfa was continued). She used 66/70 (94.3%) of her nebuliser doses over the 14 days (note that TOBI ^®^ twice daily = 4 doses via I-neb ^®^ and AZLI ^®^ could not be administered via I-neb ^®^). Her %FEV _1_ improved to 41% at day 7 and day 14, despite developing viral coryzal symptoms at day 12. With 14 days of hospital IV piperacillin/tazobactam 4.5g thrice daily and intravenous tobramycin 480mg once daily in month 11, her %FEV _1_ improved from 39% at day 1 to 41% at day 14. She received intensive physiotherapy during her in-patient stay in month 11 which may contribute to some of the %FEV _1_ improvement but received no other adjunctive therapies (e.g. corticosteroid) during all treatment courses. All her inhaled therapies were continued during dual intravenous antibiotic courses.

During the follow-up year, only Psae (Liverpool epidemic strain sensitive only to colistin) was cultured from all eight sputum samples. During the subsequent year, only Psae (Liverpool epidemic strain sensitive only to colistin) was cultured from all nine sputum samples.

## Discussion

In this case, %FEV _1_ improvement following acute treatment of exacerbations with dual inhaled antibiotics at home (mean 3.5% over two courses) was somewhat higher than with dual intravenous antibiotics (mean 0.5% over four courses), despite similar baseline %FEV _1_ and one of the intravenous antibiotics courses was delivered in hospital. The %FEV _1_ improvement also occurred despite severe background lung disease (high resolution CT in month eight showed extensive bronchiectasis and baseline %FEV _1_ was ~40%).

Although she reported symptomatic improvement during her first dual inhaled antibiotics course, we did not formally measure symptomatic responses to treatments with a validated tool. The sample size is too small for null hypothesis significance testing, and regression to the mean is potentially a threat to our results. Our results are nonetheless intriguing and suggest that dual inhaled antibiotics could potentially have a role in treating exacerbations among adults with CF and chronic Psae infection. With the increasing number of inhaled anti-pseudomonal antibiotics available, e.g. nebulised levofloxacin ^14^, different combinations of concomitant inhaled antibiotics can be used in the future for synergistic effect.

Like all medications, there are adverse events associated with inhaled antibiotics. There is a case report of acute respiratory distress syndrome potentially due to inhaled colistin ^15^. However, localised adverse events with inhaled antibiotics are usually mild, e.g. bronchoconstriction which tends to resolve spontaneously within hours or can be controlled by pre-dosing with nebulised bronchodilator ^11^.

High adherence to inhaled therapies probably contributed to the good clinical response from dual inhaled antibiotics observed in this case. Real-world adherence with long-term inhaled antibiotics among adults with CF is only 35–50% ^16, 17^. Someone who is already struggling with a single inhaled antibiotic is unlikely to cope with dual inhaled antibiotics, thus may derive less benefit. However, adherence to short-term drug regimen tends to be higher ^18^. Adults with CF might be able to summon adequate self-regulation during a 14-day dual antibiotics course to really focus on their nebuliser use ^19^.

In conclusion, the %FEV _1_ improvements observed in this case report provide anecdotal evidence that dual inhaled antibiotics could potentially be a treatment option for exacerbations among adults with CF and chronic Psae infection. Given the lack of good quality evidence regarding optimum exacerbations treatments and the theoretical advantages of using inhaled antibiotics, this warrants further investigations.

## Consent

Written informed consent for publication of her clinical details was obtained from the patient.

## Data availability

*No data is associated with this article.*
