# Supplementary material for: Case Report: Dual nebulised antibiotics among adults with cystic fibrosis and chronic Pseudomonas infection
Source: F1000Res. 2018 Feb 28;6:2079. Originally published 2017 Dec 1. [Version 2] doi: 10.12688/f1000research.13298.2 (PMC5832909; doi:10.12688/f1000research.13298.2)
Supplement: Peer review history

# Peer review: Referee response for version 2

**DOI:** 10.5256/f1000research.15356.r32187

**Published:** 2018-03-20

**Authors:** Theodore G. Liou

**Affiliations:** 1 Department of Internal Medicine, University of Utah, Salt Lake City, UT, USA

The case report suggests that dual inhaled antibiotics are feasible as an option for treatment. There were two treatment courses with dual antibiotics using aztreonam and tobramycin on both occasions. One was associated with a 5 percentage point increase in FEV1 by the end of treatment while the other was associated with a more modest 2%. There were 4 courses of intravenous antibiotics: 1-2) piperacillin/tazobactam

 + colistin with a 2 percentage point increase with course 1 and a 2 percentage point decrease with course 2; 3) piperacillin/tazobactam + colistin with a 0 percentage point increase; and 4) piperacillin/tazobactam + tobramycin with a 2 percentage point increase.

 The response to the first course of dual inhaled antibiotics was the largest. Unfortunately, it is difficult to make more of the results in terms of significance testing, as the authors note.

 Overall, the case report suggests that it is feasible to use dual inhaled antibiotics for treatment of worsening CF. Clinicians may be helped by having this possibility pointed. out.

 Colistin is misspelled as colostin in the 2nd sentence, 4th paragraph of the section entitled Case report.

I have read this submission. I believe that I have an appropriate level of expertise to confirm that it is of an acceptable scientific standard.

# Peer review: Referee response for version 2

**DOI:** 10.5256/f1000research.15356.r31267

**Published:** 2018-03-14

**Authors:** Dilip Nazareth, Freddy Frost

**Affiliations:** 1 Liverpool Heart and Chest Hospital, Liverpool, UK

We are happy with the amendments and would recommend publication.

We have read this submission. We believe that we have an appropriate level of expertise to confirm that it is of an acceptable scientific standard.

# Response

**Published:** 2018-03-14

**Authors:** Zhe Hui Hoo

**Affiliations:** University of Sheffield, UK

We thank Drs Frost and Nazareth for the positive review.

# Peer review: Referee response for version 2

**DOI:** 10.5256/f1000research.15356.r31266

**Published:** 2018-03-01

**Authors:** Tim Lee

**Affiliations:** 1 Regional Paediatric Cystic Fibrosis Unit, Leeds Teaching Hospitals NHS Trust, Leeds, UK

I thank the authors for revising the manuscript. My previous comments have been addressed and I support publication.

I have read this submission. I believe that I have an appropriate level of expertise to confirm that it is of an acceptable scientific standard.

# Response

**Published:** 2018-03-01

**Authors:** Zhe Hui Hoo

**Affiliations:** University of Sheffield, UK

We thank Dr Lee for the very prompt review.

# Peer review: Referee response for version 1

**DOI:** 10.5256/f1000research.14431.r29543

**Published:** 2018-02-08

**Authors:** Theodore G. Liou

**Affiliations:** 1 Department of Internal Medicine, University of Utah, Salt Lake City, UT, USA

This is a case report of a patient with CF during a 6 month period during which she was treated with multiple antibiotics in several regimens that seemed to differ with each treatment episode.

 The introduction is well written and informative. The case history itself raises many questions. There are fewer details of the patient’s baseline history than desired. The lack of a specific age is not typical for case reports. It would be nice to know whether her genotype was F508del homozygous or heterozygous with a second, preferably identified, mutation. The report of high adherence of the patient is open to doubt because of the poor track record of clinicians detecting non-adherence in an accurate way, and confirmation of the degree of adherence, perhaps with a medication possession report, would be helpful.

 The key portion of the history detailing treatments with various antibiotics, regimens and dosing routes is interesting but lacks some key details. It is not entirely clear what triggered the more intensive 6 months. There is reference to symptoms and signs often associated with acute exacerbations, the introduction seems to focus at least in part on exacerbations, and the discussion refers to “acute treatment of exacerbations,” but the patient’s course is not described in a way as to identify when pulmonary exacerbations were specifically diagnosed or treated. This detracts from helping readers understand the circumstances for which different treatments were initiated.

 In the discussion, the authors discuss the possible effects of dual inhaled antibiotics, but I find it difficult to associate such use with a superior outcome even on an anecdotal basis because many drugs and regimens were employed in response to what seems to have been multiple clinical situations during the 6-month case history period.

 I think the report could be improved by providing more patient history prior to the 6-month period of focus, better assessment of her level of adherence, clear definition of pulmonary exacerbation as used in this patient’s case and clearer description of triggers for different treatments as well as more precise descriptions of outcomes. An additional outcome to consider including would be the results of subsequent bacterial cultures and any changes in resistance patterns.

 A study of dual inhaled antibiotics compared to intravenous would be of interest. However, there are likely to be many barriers and difficulties developing a generally usable protocol. Some mention of these barriers might be helpful to explain why such a study has not been done.

 Finally, to help readers in areas of the world where trade names are not the same or the medications are not available, Promixin, Azli, Tobi, Tazocin and Coly-Mycin should be identified by their generic names in addition to the trademarked names. It would be better, I think, if they were referred to by their generic names in preference to the trade names.

I have read this submission. I believe that I have an appropriate level of expertise to confirm that it is of an acceptable scientific standard, however I have significant reservations, as outlined above.

# Response

**Published:** 2018-02-08

**Authors:** Zhe Hui Hoo

**Affiliations:** University of Sheffield, UK

We thank Dr Liou for the review and we will iterate the case report taking into account the comments.

In Sheffield whereby we only provide care for around 200 adults with CF, we only have a female adult with her exact age (in years) and her F508del/Class I mutation. Thus, to maintain the anonymity of the patient (which was the basis on which she provided consent), we could only provide an approximate age and specify that she was heterozygous for F508 mutation in the first sentence of the 'case report' section. In any case, whether the patient is 30 years of age or 33 years of age (which is the age range we consider to sit within 'early thirties') is irrelevant to the conclusion of the case report or to the interpretation of her FEV1. By stating that she has a Class II mutation (i.e. F508del) and Class I mutation in the first sentence of the 'case report' section, we have also specified that she has 'severe genotype' according to the definition of Castellani et al. J Cyst Fibros 2008, 7:179-196.

In Sheffield, we measure nebuliser adherence using I-neb (as specified in the second sentence of the 'case report' section). Electronic data capture is generally considered the 'gold standard' measure for adherence and MPR is not a "better assessment of adherence" compared to electronic data capture. The study by Siracusa et al. J Cyst Fibros 2015;14:621-6 have demonstrated that MPR data may be unreliable. In that study, ivacaftor adherence according to electronic data capture was only around 60% but MPR suggested adherence of 84%. There was also no correlation between adherence data captured using electronic data capture and MPR. Since we have provided EDC adherence data, we do not think MPR data will add further information regarding her adherence. 

We will clarify the trigger for the more intensive treatment period and clarify which of the treatment was used to treat an acute exacerbation. We will also provide results for her subsequent bacterial cultures and any changes in resistance patterns.

A small study using dual nebulised antibiotics has been done in 1985, as identified by the 2012 Cochrane review. We agree that any study for the treatment of pulmonary exacerbation is challenging. The STOP program in the US appears to be making breakthroughs in starting to define best practices in the treatment of pulmonary exacerbations, suggesting that such studies are difficult but not impossible to execute.

# Peer review: Referee response for version 1

**DOI:** 10.5256/f1000research.14431.r30297

**Published:** 2018-01-30

**Authors:** Dilip Nazareth, Freddy Frost

**Affiliations:** 1 Liverpool Heart and Chest Hospital, Liverpool, UK

Mann *et al *report an interesting case demonstrating that inhaled antibiotics may be useful for the treatment of acute pulmonary exacerbations in CF. The authors accurately describe the background and much of the evidence relating to the use of inhaled antibiotics in acute exacerbations including the Cochrane review of 2012. However, the Cochrane review preceded the most recent clinical trial in this field. The study demonstrated and discussed both pulmonary and renal benefits for nebulized tobramycin in the treatment of exacerbations and the authors should consider including it in the introduction. _1_

 In the fourth paragraph it should be clarified whether “typically one inhaled antibiotic is used at a time” refers to the routine practice in the chronic setting or previous studies in the acute setting.

 The case itself is well described however the authors may wish to consider including some relevant extra information. Firstly, had the patient cultured any other organisms aside from *P. aeruginosa  *in their sputum in the preceding 12 months? Secondly, were all the treatments completed at home or in the inpatient setting? Differing settings with increased physiotherapy input as an inpatient could theoretically account for some of the differences. Finally, were other adjunctive therapies such as oral corticosteroids used in the management of each exacerbation.

 The discussion is balanced and recognises the limitations of the case as well as potential adverse events related to nebulised therapy. Overall, this is an interesting case worthy of publication and we wholeheartedly agree with the authors that this is a management strategy that warrants further investigation.

We have read this submission. We believe that we have an appropriate level of expertise to confirm that it is of an acceptable scientific standard, however we have significant reservations, as outlined above.

## References

[1] Al-AloulMNazarethDWalshawM: Nebulized tobramycin in the treatment of adult CF pulmonary exacerbations. *J Aerosol Med Pulm Drug Deliv*.2014;27(4) : 10.1089/jamp.2013.1055 299-305 10.1089/jamp.2013.1055 24219814

# Response

**Published:** 2018-01-30

**Authors:** Zhe Hui Hoo

**Affiliations:** University of Sheffield, UK

We thank Drs Frost and Nazareth for the review and very useful comments which will guide our revision of the case report.

We will include 2014 pilot study of nebulised vs IV tobramycin in the 'Introduction'. We will also alter the 4th paragraph of the 'Introduction' to clarify that the first sentence was referring to inhaled antibiotics in chronic settings whilst the second sentence referred to inhaled antibiotics in acute settings.

We will include the extra information relating to microbiology results, clarify the treatment setting (all treatments were at home except for the last course of IV antibiotics in month 11) and clarify the use of any adjunctive therapies.

# Peer review: Referee response for version 1

**DOI:** 10.5256/f1000research.14431.r29445

**Published:** 2018-01-04

**Authors:** Tim Lee

**Affiliations:** 1 Regional Paediatric Cystic Fibrosis Unit, Leeds Teaching Hospitals NHS Trust, Leeds, UK

This is a useful case report highlighting the potential for treating certain pulmonary exacerbations in people with cystic fibrosis with inhaled antibiotics. The background is well written. References are appropriate and balanced.

 The case is well described and the adherence data prior to the exacerbations is helpful. The clinical response in terms of FEV1 is well reported and favourable by comparison to response to iv therapy.

 Paragraph 2 of the "Case Report" section should in my view more clearly specify that the colomycin 2MU three times a day was given **intravenously **rather than nebulised alongside the intravenous Tazocin for 14 days in month 1 and month 4, given the overall title of the case report and the similar iv/nebulised dosages for colomycin. It would also be instructive to know whether the intravenous antibiotics were administered at home or in hospital.

 In paragraphs 3 and 4 of the "Case Report" section, it would be useful to know if the subject was also taking nebulised dornase alpha during this period, i.e. was she achieving six nebulised therapies per day? Also, if available, adherence measures during these 2 week pulses of inhaled dual antibiotic therapies would be useful, as many clinicians do have concerns that this intensity of nebulised medications may be difficult to achieve for many people with CF, despite the good clinical response seen here.

 The conclusions, acknowledgements of limitations, are all well written and appropriate. In summary this case report is valuable and will be of interest to the CF community. I support publication but would recommend the clarifications I have suggested are considered.

I have read this submission. I believe that I have an appropriate level of expertise to confirm that it is of an acceptable scientific standard, however I have significant reservations, as outlined above.

# Response

**Published:** 2018-01-04

**Authors:** Zhe Hui Hoo

**Affiliations:** University of Sheffield, UK

We thank Dr Lee for the review and very useful comments which will guide our revision of the case report.

We will clarify in Paragraph 2 (and 4) of the "Case Report" section that colomycin 2MU thrice daily was given intravenously, and likely for tobramycin 480mg once daily (in paragraph 4).

We will also clarify the Paragraphs 2 and 3 of the "Case Report" section that once daily nebulised dornase alfa was continued throughout the dual nebulised antibiotics; and report the total number nebuliser doses taken during the 2 week pulses of dual inhaled dual antibiotic therapies.
